# Forchlorfenuron Disrupts SEPT9_i1 Filaments and Inhibits HIF-1

**DOI:** 10.1371/journal.pone.0073179

**Published:** 2013-08-19

**Authors:** Dikla Vardi-Oknin, Maya Golan, Nicola J. Mabjeesh

**Affiliations:** Prostate Cancer Research Laboratory, Department of Urology, Tel Aviv Sourasky Medical Centre, Sackler Faculty of Medicine, Tel Aviv University, Tel Aviv, Israel; Innsbruck Medical University, Austria

## Abstract

Forchlorfenuron (FCF) is a synthetic plant cytokinin that has been shown to alter yeast and mammalian septin organization. Septins are a highly conserved family of GTP-binding cytoskeletal proteins. Mammalian septins are involved in diverse cellular processes including tumorigenesis. We have been studying the interaction between septin 9 isoform 1 (SEPT9_i1) and hypoxia inducible factor-1α (HIF-1α), the oxygen regulated subunit of HIF-1. HIF-1 is a key transcription factor in the hypoxic responses pathway, and its activation has been observed in carcinogenesis and numerous cancers. SEPT9_i1/HIF-1α interaction plays an important role in upregulation of HIF-1 transcriptional activity by preventing HIF-1α’s ubiquitination and degradation leading to increased tumor growth and angiogenesis. We tested the hypothesis whether FCF affects SEPT9_i1 filamentous structures and consequently HIF-1 pathway in cancer cells. We showed that FCF suppresses tumorigenic properties, including proliferation, migration and transformation, in prostate cancer cells. FCF did not alter SEPT9_i1 steady state protein expression levels but it affected its filamentous structures and subcellular localization. FCF induced degradation of HIF-1α protein in a dose- and time-dependent manner. This inhibition was also shown in other common cancer types tested. Rapid degradation of HIF-1α protein levels was accompanied by respective inhibition in HIF-1α transcriptional activity. Moreover, HIF-1α protein half-life was markedly decreased in the presence of FCF compared with that in the absence of FCF. The FCF-induced degradation of HIF-1α was mediated in a significant part via the proteasome. To the best of our knowledge, this is the first demonstration of specific manipulation of septin filaments by pharmacological means having downstream inhibitory effects on the HIF-1 pathway.

## Introduction

Forchlorfenuron (FCF; 1-(2-chloro-4-pyridyl)-3-phenylurea, 4PU300), also known as PESTANAL^®^, is a small synthetic molecule that is currently utilized in agriculture as growth hormone. FCF promotes cell division and is involved in cell growth and differentiation. It has a cytokinesis inhibitor effect in higher concentrations [1]. It was incidentally found that FCF causes rapid and reversible deformation of septin filament on the bud neck in yeast [2]. In mammalian cells, FCF suppresses normal septin dynamics and stabilizes septin polymers, resulting in cell morphology changes, mitotic defects, and decreased cell migration [3]. Stabilization of septin filaments by FCF reduces the turnover rate of septin filaments and thus disrupts their proper function. FCF directly and specifically alters septin assembly in mammalian cells without affecting either actin or tubulin polymerization [3].

Mammalian septins are a family of GTP-binding proteins evolutionarily conserved with roles in multiple core cellular functions. The increasingly accumulating data from studies on mammalian septins suggest that septin heteromeric complexes provide higher order structures that can act as scaffolds of docking sites for other proteins important in key cellular processes. There are 13 genes encoding both ubiquitous and tissue-specific septins [4]. *SEPT9* has been identified as a potential oncogene, and its amplification and/or overexpression was observed in several carcinomas, including breast [5–7], ovarian [8,9], head and neck [10,11] and prostate [12].

SEPT9_i1, a product of transcript *SEPT9_v1* that encodes isoform 1, was identified as a positive regulator in the hypoxic pathway. SEPT9_i1 interacts with hypoxia-inducible factor 1α (HIF-1α), the oxygen-regulated subunit of HIF-1, which mediates adaptive responses to hypoxia. The interaction with SEPT9_i1 is specific to HIF-1α, but not to HIF-2α. It increases HIF-1α protein stability as well as HIF-1 transcriptional activity, leading to enhanced proliferation, tumor growth and angiogenesis [12]. HIF-1 is a heterodimer composed of HIF-1α and HIF-1β subunits [13]. The abundance and activity of the HIF-1α subunit are regulated by O_2_-dependent hydroxylation [14]. Proline hydroxylation targets HIF-1α for ubiquitination by the von Hippel-Lindau ligase complex and subsequent proteasomal degradation [14,15] whereas asparagine hydroxylation blocks interaction of HIF-1α with the coactivator p300 [16,17]. Under hypoxic conditions, hydroxylation is inhibited and HIF-1α rapidly accumulates and translocates to the nucleus where it dimerizes with HIF-1β. HIF-1 binds to hypoxia response elements (HRE) to drive the transcription of numerous genes that are important for adaptation and survival under hypoxia, including glycolytic enzymes, the glucose transporters Glut-1 and Glut-3, endothelin-1 (ET-1), vascular endothelial growth factor (VEGF), carbonic anhydrase IX (CA-IX), and erythropoietin [18].

SEPT9_i1 increases HIF-1α protein expression levels by decreasing HIF-1α ubiquitination and degradation via the O_2_-independent pathway mediated by RACK1 (receptor of activated protein kinase C 1) E3 ligase [19]. Since FCF affects septin organization and dynamics in mammalian cells, and given that SEPT9_i1/HIF-1α interaction has a major role in the activation of the HIF-1 pathway, we tested the hypothesis whether FCF affects SEPT9_i1 filamentous structures and consequently affects the HIF-1 pathway in cancer cells.

## Materials and Methods

### Cell culture and hypoxia treatment

All human cell lines (PC-3, LNCap, MCF-7, HCT116 and MDA-MB -231) were purchased from the American Type Culture Collection (ATCC). Human prostate cancer PC-3 and LNCaP cells were maintained in RPMI 1640, human breast carcinoma MCF-7 and MDA-MB-231cells were maintained in DMEM, and human colon cancer HCT116 cells were maintained in modified McCoy’s medium. All media were supplemented with 10% FCS and antibiotics. Cells were cultured at 37°C in a humidified atmosphere and 5% CO_2_ in air. For hypoxic exposure, cells were placed in a sealed modular incubator chamber (Billups-Rothenberg, Del Mar, CA) flushed with 1% O_2_, 5% CO_2_, and 94% N_2_ and then cultured at 37°C.

### Antibodies and reagents

The following primary antibodies were used: rabbit polyclonal antibody to SEPT9_i1 previously produced and characterized [12], mouse monoclonal anti-HIF-1α (BD Biosciences, San Diego, CA), and mouse monoclonal anti-α-tubulin (Sigma-Aldrich, St. Louis, MO). Secondary antibodies were horseradish peroxidase conjugated (Jackson ImmunoResearch, West Grove, PA) for Western blotting and Alexa Flour 594 donkey anti-mouse and 488 donkey anti-rabbit (Invitrogene, Carlsbad, CA) for immunofluorescence. MG-132, cycloheximide (CHX) and epoxomicine were purchased from Sigma-Aldrich.

### FCF Treatment

FCF (Sigma Aldrich) at purity of 99.9 area % by HPLC assay was dissolved in DMSO to yield 500 mM stock solution. The stock was diluted in the appropriate media to reach the indicated concentrations, and DMSO was added to reach a final concentration of 0.08% in all conditions, including vehicle controls.

### Cell proliferation assay

Cells were seeded in 96-well-plates (1000 cells/well) in a volume of 200 μL for cell proliferation assay using 3-bis-(2-methoxy-4-nitro-5 sulfenyl)-(2H)-tetrazolium-5-carboxanilide (XTT) kit (Biological Industries Ltd., Israel). On the next day, the cells were treated with different concentration of FCF and cultured under normoxic conditions. XTT reagent was added in at least triplicates for each time point and processed according to the manufacturer’s instructions.

### Cell cytotoxicity assay

Cells were seeded in 96-well plates at a density of 1,000–2,000 cells/well in 100 μl medium. On the following day, the cells were treated with increasing concentrations of FCF (in triplicates) for 3 days and processed for sulforhodamine B (SRB) cytotoxicity assay as originally described by Skehan et al. [20].

### Wound healing assay

Cells were grown to confluence in 6-well plates. After 24 h, the monolayer was scratched using a 200 μL sterile plastic pipette tip and washed twice with complete medium. The cells were treated with different concentration of FCF and were allowed to migrate onto the plastic surface. Five random pictures were taken for each wound immediately after a wound had been inflicted to the cell monolayer and after 2, 4, 6 and 8 h. The area of the wound was measured by using a rectangle area selection tool, and the five areas per well were averaged.

### Soft agar assay

Cells (5,000/plate) were plated in a volume of 2 ml agar (0.33%) over 1 ml of base layer agar (0.5%). They were treated with vehicle or 100 µM FCF and incubated for 4 weeks. Colonies (≥20 cells) were examined and counted under an Olympus inverted microscope using an x20 lens.

### Protein extraction and immunoblot analysis

Cells were washed with ice-cold PBS prior to harvesting. Whole cell extracts (WCE) were prepared by lysing the cells with 100 mM potassium phosphate (pH 7.8) and 0.2% Triton X-100 supplemented with protease inhibitors. Protein concentration was determined using a BCA protein assay kit (Pierce, Rockford, IL). Protein extracts were analyzed by SDS-PAGE and immunoblotted with the antibodies as displayed in the Figures. Between 30–60 μg of protein were loaded in each lane.

### Protein stability assay

Cells were plated into 6-well plates and grown to 70% confluence. They were treated with FCF for 4 h under normoxic conditions. Cycloheximide was added to the cells at a final concentration of 10 µg/ml for the indicated times. The cells were lysed and subjected to immunoblotting as described above.

### Co-immunoprecipitation assay

Cells were grown in 6-cm plates and then treated with different concentration of FCF or vehicle and incubated either under normoxic or hypoxic conditions for 4 h. They were harvested and lysed with 20 mM Na-HEPES (pH 7.5), 0.5% NP40, 0.1 M NaCl, 2 mM EDTA and 10% glycerol supplemented with protease inhibitors. Lysates were incubated overnight with the selected antibody at 4°C. Protein G-Sepharose beads (Sigma-Aldrich Co., St. Louis, MO) were then added and the mixture was further incubated at 4°C for 1 h. After the beads were thoroughly washed with lysis buffer, SDS-sample buffer x2 was added and the samples were boiled for 10 min. Immunoprecipitates were separated on SDS-PAGE and analyzed by Western blotting.

### Reporter gene assay

HIF-1 HRE-dependent luciferase (firefly) activity was performed using the pBI-GL construct (pBI-GL V6L) containing six tandem copies of the *VEGF* HRE and SV40-dependent renilla luciferase reporter plasmid (Promega, Madison, WI). HIF-2 transcriptional activity was measured using pGL2 construct containing P2 PTHrP promoter [21]. Cells were grown in 6-well plates and then transiently transfected in triplicate with 1µg DNA of reporter plasmid. Twenty-four h after transfection, the cells were treated with FCF and incubated either under normoxic or hypoxic conditions for 16 h. Cells were harvested with 100 mM potassium phosphate (pH 7.8) and 0.2% Triton X-100 supplemented with protease inhibitors. Luciferase enzymatic activity was measured using luciferase reporter assay or a dual luciferase reporter assay (Promega, Madison, WI) following the manufacturer’s instructions. Luminescence was measured by a BMG Labtechnologies LUMIstar Galaxy luminometer.

### RNA purification and quantitative real-time PCR analyses

Total RNA was extracted from cells using NucleoSpin RNA II kit (Macherey-Nagel, Duren, Germany) following the manufacturer’s instructions. One microgram of total RNA was reverse transcribed into cDNA using VersoTM cDNA kit (ABgene, Epsom, United Kingdom) using anchored oligo (dT) as first-strand primer. Quantitative real-time PCR (qRT-PCR) analyses were performed to determine the expression of *HIF-1α, Glut-1*, *ET-1* and for cyclophilin B as an internal control. qRT-PCR reactions were done in duplicate using LightCycler FastStart DNA Master SYBR Green I (Roche Applied Science, Mannheim, Germany). The PCR reaction was performed at a total volume of 10 μl, using 3 mM of MgCl_2_ and 0.5 μM of each primer. The expression of the *hif-1α, Glut-1* and *ET-1* genes was normalized to cyclophilin B expression levels.

### Immunofluorescence

Cells were plated on 12-mm glass coverslips (Fisher Scientific, Pittsburgh, PA) inserted into 6-well plates and allowed to attach overnight. Cells were fixed with cold methanol for 10 min and permeabilized by cold acetone for 3 min room temperature. Blocking was done in 1% BSA/10% normal donkey serum/PBS for 30 min at room temperature. Cells were subsequently incubated with primary antibodies to HIF-1α (1:50) and polyclonal rabbit anti-SEPT9 (1:250) diluted in primary antibody dilution buffer (Biomeda, Foster City, CA) for 2 h at room temperature. Secondary antibodies (Alexa Flour 594 donkey anti-mouse and 488 donkey anti-rabbit diluted 1:300 in PBS) were incubated for 1 h in the dark and DAPI was added for 3 min at room temperature. Samples were mounted onto slides using Fluorescent Mounting Medium (Golden Bridge International [GBI] Life Science Inc, Mukilteo, WA) and then examined under a Leica SP5 confocal microscope using a x63 NA1.4 lens. Laser and microscope settings were according to the manufacturer’s instructions. Identical parameters (e.g., scanning line, laser light, contrast, and brightness) were used for comparing fluorescence intensities, and 5 microscopic fields were taken from each sample.

### Statistical analysis

The experiments presented in the figures are representative of three or more independent repetitions. The data are expressed as means ± SD. Student’s *t* test was used to compare differences between particular conditions. Statistical significance was set at *P* < 0.05.

## Results

### FCF suppressed cancer cell tumorigenic properties

**Figure 1:**
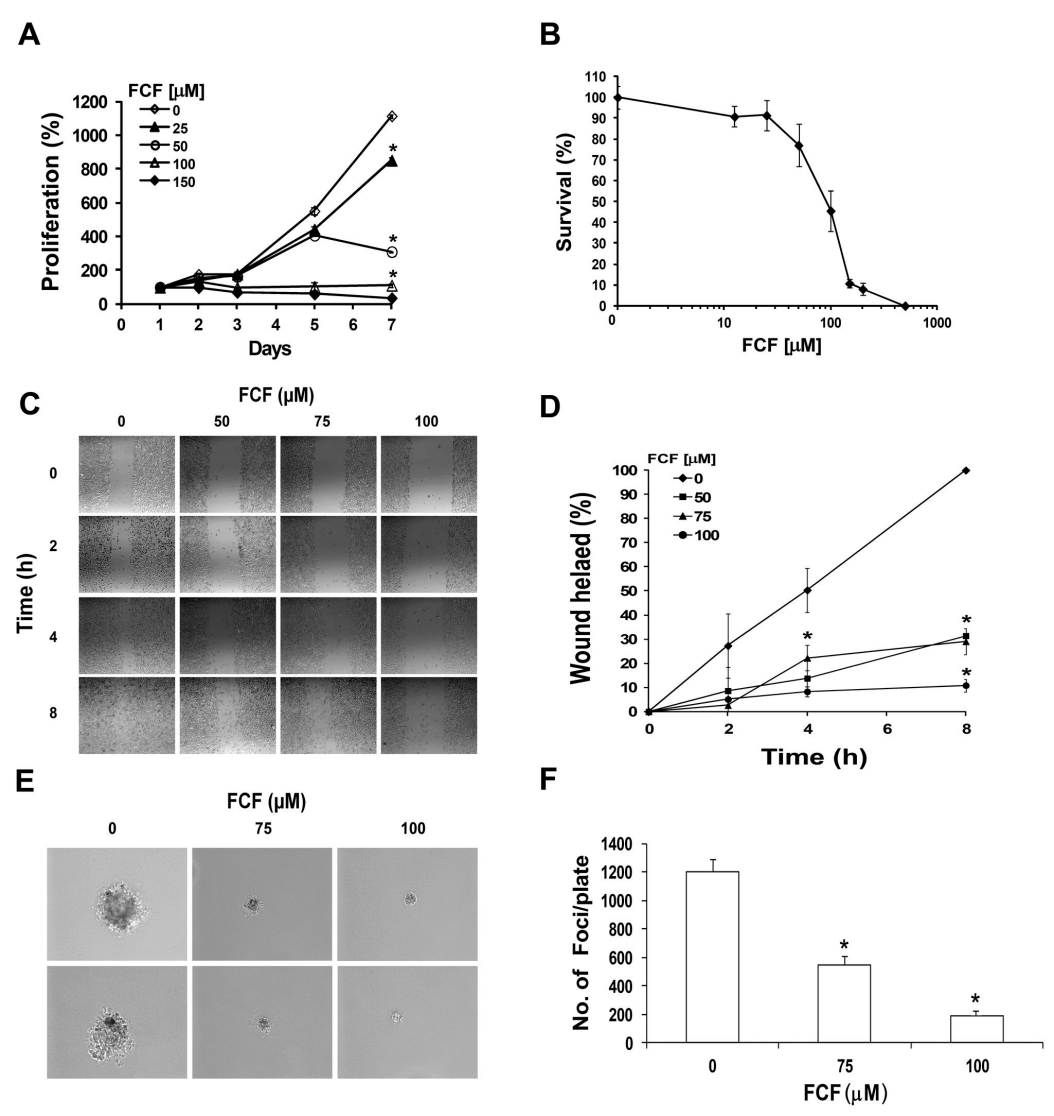
FCF inhibits cell proliferation, migration and transformation. (**A**) PC-3 cells were treated with increasing concentrations of FCF and grown under normoxic conditions. Cells were analyzed for proliferation at the indicated times using XTT assay. Proliferation was expressed as increase in percentage of the initial absorbance that was measured 24 h after seeding (100%). Growth media and treatment were changed every other day. *Points*, mean (n=3 replicates); bars, SD; *P < 0.001. This is a representative experiment out of 3 independent repetitions. (**B**) PC-3 cells were treated with FCF and grown under normoxic conditions for 72 h and processed for SRB cytotoxicity assay. Cell survival was expressed as percentage of the initial absorbance measured in vehicle (0.08% DMSO) control cells (100%). *Points*, means (n=3); bars, SD. (**C**) PC-3 cells were grown in 6-well plates to reach 90% confluence. They were then treated with FCF and grown under normoxic conditions for 16 h. The cell monolayer was scratched using a sterile 200-μl pipette tip, and the wounded cultures were watched and photographed after 0, 2, 4 and 8 h (magnification x10). (**D**) Wound healing was calculated as percentage of the wound area in vehicle-treated cells at 8 h (100%). *Points*, mean (n = 3); *bars*, SD. *P < 0.01. (**E**) PC-3 cells were grown on soft agar and treated with 0, 75 or 100 µM FCF, under normoxic conditions for 3 weeks. A representative colony from each FCF treatment is shown (Magnification x20). (**F**) A quantitative analysis of colonies for each treatment. *Columns*, means (n=2); *bars*, SD. *P < 0.01.

FCF reportedly reduced the turnover rate of human septins, thereby affecting the relative abundance of cytoplasmic and filamentous septins, which is critical for their function [3]. Altered levels of septin expression were found to correlate with tumorigenic phenotypes [5,7,22]. We therefore examined the biological effects of FCF on prostate cancer cells (PC-3). We first tested whether FCF affects cell proliferation and observed that the PC-3 cell proliferation rate was significantly decreased by FCF (Figure 1A). We also evaluated the cytotoxic effects of FCF in PC-3 cells by exposing them to increasing concentrations of FCF for 3 days and then subjecting them to SRB cytotoxicity assay (Figure 1B). The mean IC_50_ value of FCF in PC-3 cells was 90 ± 17.3 μM, as calculated from 3 independent experiments. Next, we examined whether FCF affects cell migration using a scratch wound-healing assay and showed that FCF treatment dramatically decreased cell migration in a dose-dependent manner (Figure 1C & D). Specifically, FCF inhibited cell migration by more than 70%, compared to control (Figure 1D). We tested the effects of FCF on cell transformation using a soft agar assay (Figure 1E & F). FCF-treated cells formed significantly smaller and fewer colonies in a dose-dependent manner. Specifically, FCF treatment at concentrations of 75 μM and 100 μM decreased the number of colonies by 55% and 85%, respectively (Figure 1F). Altogether, these results showed that FCF significantly suppresses tumorigenic properties of prostate cancer cells, including their proliferation, migration and transformation.

### FCF decreased HIF-1α protein expression levels and HIF-1 transcriptional activity

**Figure 2:**
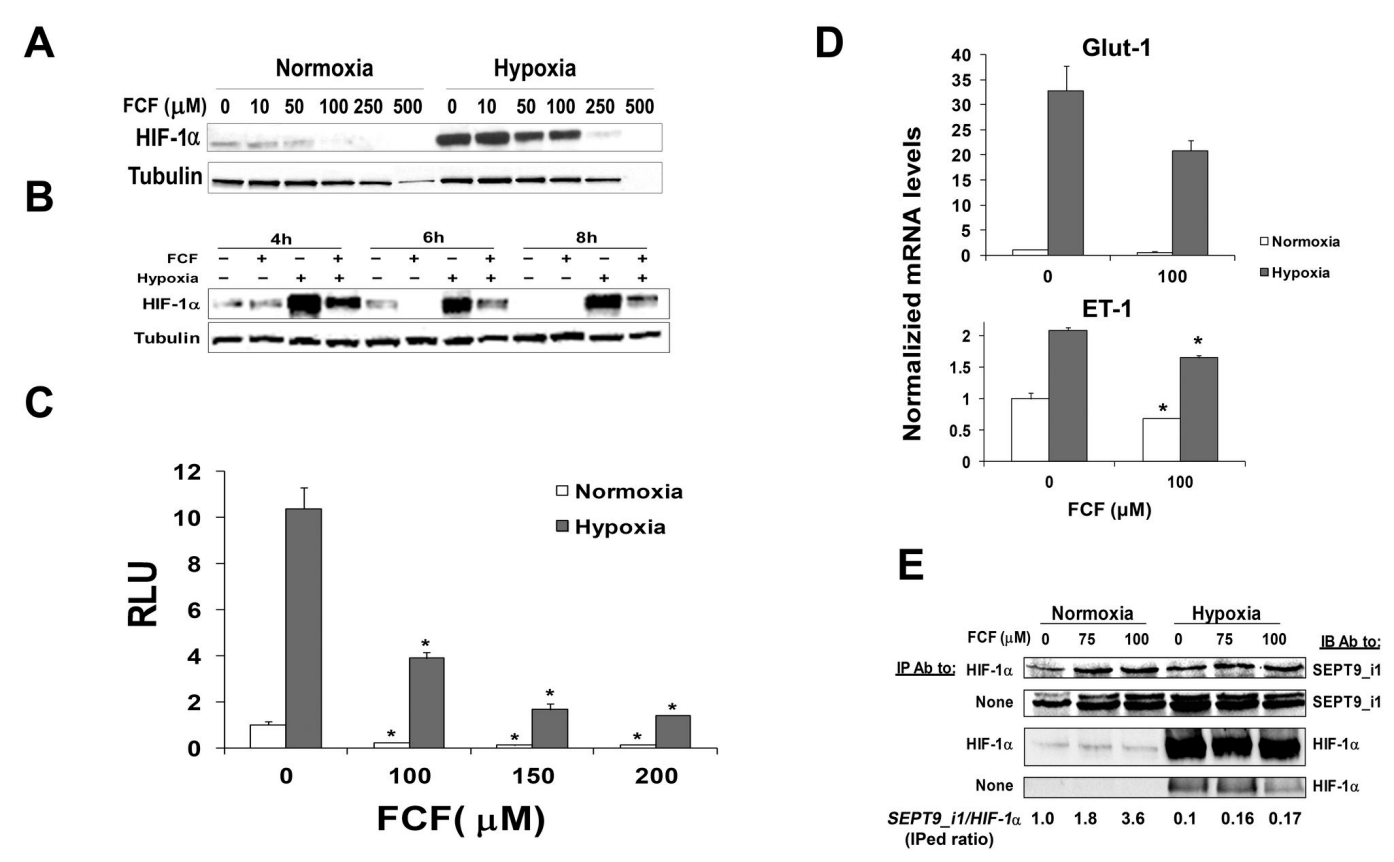
FCF decreases HIF-1α protein expression and HIF-1 transcriptional activity. PC-3 cells were treated with increasing concentrations of FCF for 6 h (**A**) or with 100 μM FCF for the indicated times (**B**) under normoxic and hypoxic conditions. Whole cell extracts were analyzed by SDS-PAGE and immunoblotted with antibodies to HIF-1α and tubulin. (**C**) PC-3 cells were transiently co-transfected with HRE-dependent firefly luciferase reporter and SV40-dependent renilla luciferase reporter plasmid. After 24 h of transfection, the cells were pretreated with vehicle or 100 µM FCF for 2 h and then grown overnight under normoxia or hypoxia. Whole cell extracts were analyzed by dual luciferase reporter assay. Relative luciferase units (RLU) represent arbitrary units of firefly luciferase activity normalized to renilla luciferase activity. Values were normalized to control vehicle at normoxia. *Columns*, mean (n = 3); *bars*, SD; *P < 0.01. (**D**) PC-3 cells were treated or not treated with 100 μM FCF for 2 h and then subjected overnight to normoxic or hypoxic conditions. Total RNA was isolated from the cells and analyzed by quantitative real-time PCR using primers for Glut-1, ET-1, and cyclophilin B as control. The results were normalized to cyclophilin B mRNA expression levels, and the mean induction of each gene was normalized to control untreated cells under normoxia. *Columns*, means (n=2); *bars*, SD; *P < 0.05. (**E**) PC-3 cells were treated with 0, 75 and 100 μM FCF for 2 h and then subjected to normoxia or hypoxia for an additional 4 h. Cellular extracts were subjected to immunoprecipitation (IP) using anti-HIF-1α antibodies and then immunoblotted (IB) with antibodies to SEPT9_i1 and HIF-1α. *None* refers to no IP, whole cell extracts only.

Since SEPT9_i1 interacts and stabilizes HIF-1α protein to increase its activity [12], we investigated whether FCF would also affect HIF-1α. First, we tested the influence of FCF on HIF-1α protein levels. PC-3 cells were pre-incubated with increasing concentrations of FCF and subjected to normoxic or hypoxic conditions (Figure 2A). We observed a significant reduction in HIF-1α protein expression levels in correlation with FCF concentration under conditions of both normoxia and hypoxia. Noteworthy, the decline in HIF-1α was more pronounced in normoxia than in hypoxia. FCF over 200 μM concentration seemed to be toxic for the cells, as seen by cell death and lower tubulin levels (Figure 2A). We also examined the time-course of the FCF effect on HIF-1α protein expression levels by treating PC-3 cells with FCF and then incubating them under normoxia or hypoxia for various times (Figure 2B). Since the expression of HIF-1α protein under normoxia could be influenced by cell density as shown previously by Zhong et al. [23] a normoxia control was used for each time point. FCF treatment for 4-6 h yielded the most significant reduction in HIF-1α protein levels under both conditions. We tested whether FCF also affects HIF-1 transcriptional activity. PC-3 cells were transiently co-transfected with a reporter plasmid containing the firefly luciferase gene under the control of HRE from the *VEGF* promoter and SV40-dependent renilla luciferase reporter plasmid. Cells were treated with FCF and then incubated overnight under normoxic or hypoxic conditions (Figure 2C). FCF significantly inhibited HIF-1 transcriptional activity by more than 50% under normoxia and hypoxia, compared to the control. The expression of two additional HIF-1-target genes, *Glut-1* and *ET-1*, was also inhibited by FCF as measured by real-time qRT-RCR (Figure 2D). Of note, the *ET-1* transcript was increased by two folds under hypoxia while *Glut-1* was increased by more than 30-fold. HIF-target genes are differentially regulated by hypoxia depending on tight coordination of expression under specific conditions [24]. To better understand the mechanism of action of FCF, we tested whether it affects HIF-1α interaction with SEPT9_i1. We have previously studied the specificity of the interaction between HIF-1α and SEPT9_i1 including the identification of the interacting regions [12,19]. PC-3 cells were treated with FCF, incubated under normoxic or hypoxic conditions and subjected to immunoprecipitation with HIF-1α antibodies (Figure 2E). FCF increased HIF-1α-SEPT9_i1 interaction by almost 3-fold under normoxia as quantified by densitometry. The interaction of HIF-1α with SEPT9_i1 was much weaker under hypoxia, but it also increased by about 2-fold in the presence of FCF (Figure 2E). Collectively, FCF reduced HIF-1α protein expression and inhibited HIF-1 transcriptional activity, while it increased HIF-1α-SEPT9_i1 interaction.

### FCF accelerated HIF-1α degradation through the proteasome

**Figure 3:**
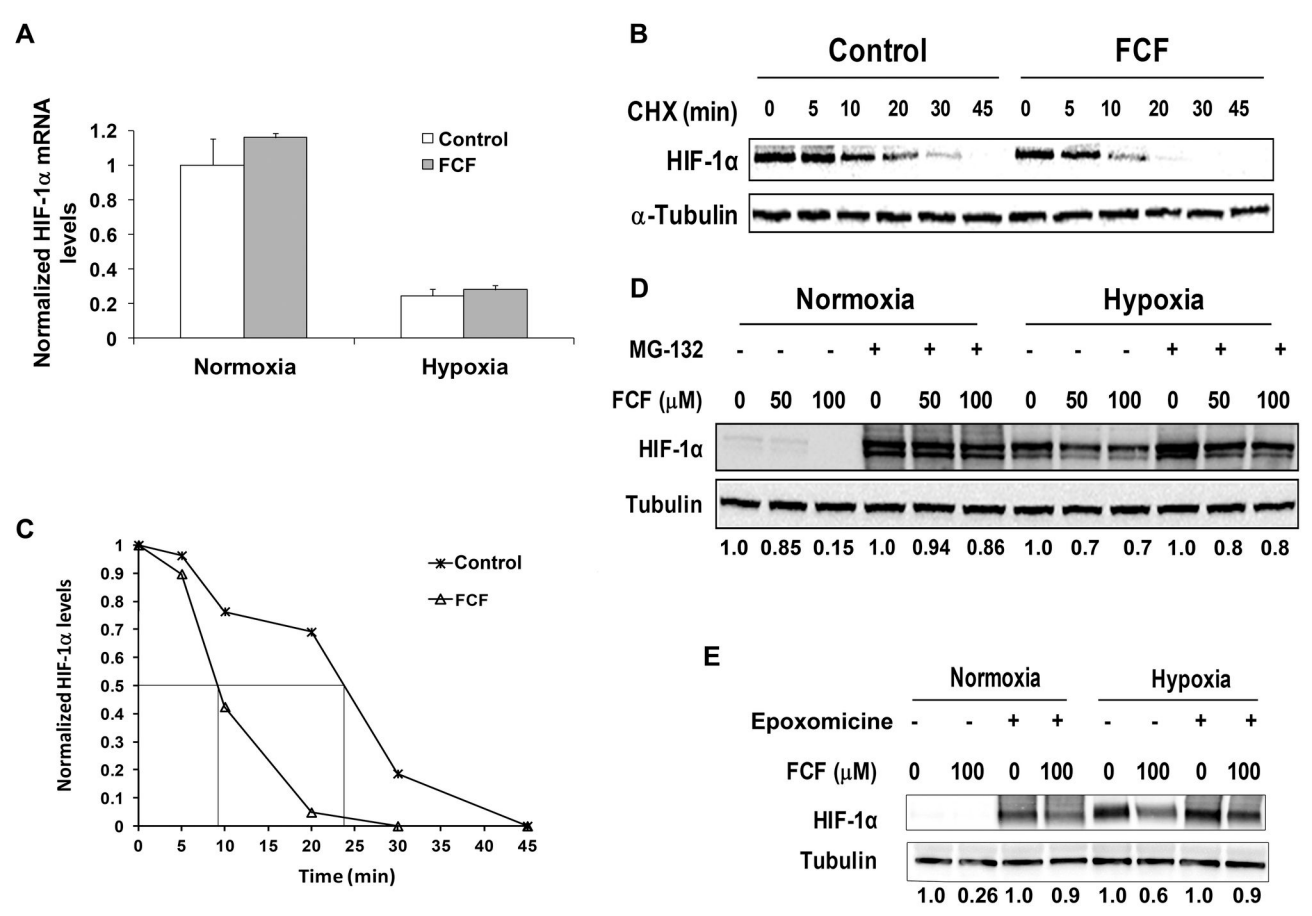
FCF affects HIF-1α expression on the posttranslational level. (**A**) PC-3 cells were pretreated with 100 μM FCF for 2 h and then subjected overnight to normoxic or hypoxic conditions. Total RNA was isolated and reverse transcribed into cDNA. Quantitative real-time PCR analysis was done using primers for HIF-1α and cyclophilin B as control*. Columns*, means (n = 2); *bars*, SD. (**B**) PC-3 cells were treated with vehicle (control) and 100 μM FCF for 4 h under normoxia, and then CHX was added at a final concentration of 10 μg/ml for the indicated times. Whole cell extracts were analyzed by SDS-PAGE and immunoblotted with antibodies to HIF-1α and tubulin. (**C**) Densitometric quantification of HIF-1α levels in (**B**) normalized to tubulin. Fifty% decrease of HIF-1α levels by FCF is delineated in grey lines. This is a representative experiment out of 3 independent repetitions. PC-3 cells were treated with FCF in the presence of 10 µM MG-132 (**D**) or 1 µM epoxomicine (**E**) for 2 h and then grown under normoxia and hypoxia for 4 additional h. Whole cell extracts were prepared, analyzed by SDS-PAGE and immunoblotted with antibodies to HIF-1α and tubulin. Densitometric quantification of HIF-1α/tubulin levels normalized to control at each condition is outlined under the respective lanes.

To determine whether FCF affects HIF-1α at the transcriptional level or posttranslationally, we first looked for changes in HIF-1α mRNA expression levels. PC-3 cells were treated or not treated with FCF and then incubated overnight under normoxic and hypoxic conditions. The transcript levels of HIF-1α were analyzed by quantitative real-time PCR (Figure 3A). Since FCF did not alter HIF-1α mRNA levels, we next examined FCF effects on HIF-1α protein stability. PC-3 cells were treated or not treated with FCF and then exposed to CHX, an inhibitor of protein synthesis, for the indicated times (Figure 3B). The HIF-1α protein levels in these cells decreased by 50% within 24 min of exposure to CHX in the untreated cells, whereas they were decreased by 50% after only 9 min in the FCF-treated cells (Figure 3C). These results showed that FCF shortened the HIF-1α half-life by more than 2-fold. To examine whether FCF causes HIF-1α protein degradation via the proteasome, cells were treated with FCF in the absence or presence of the proteasome inhibitors MG-132 (Figure 3D) and epoxomicine (Figure 3E). The inhibitory effect of 100 μM FCF on HIF-1α protein levels was almost restored (up to 80-85% as quantified by densitometry) to control levels by inhibition of the proteasome under both normoxia and hypoxia (Figure 3D & E). Of note, the band of HIF-1α is usually broad and consists of multiple species of modified forms of HIF-1α, such the phosphorylated form [25]. Taken together, FCF suppressed HIF-1α protein expression and HIF-1 transcriptional activity in a significant part by increasing HIF-1α protein degradation through the proteasome.

### FCF altered SEPT9_i1 organization and sub-cellular localization

**Figure 4:**
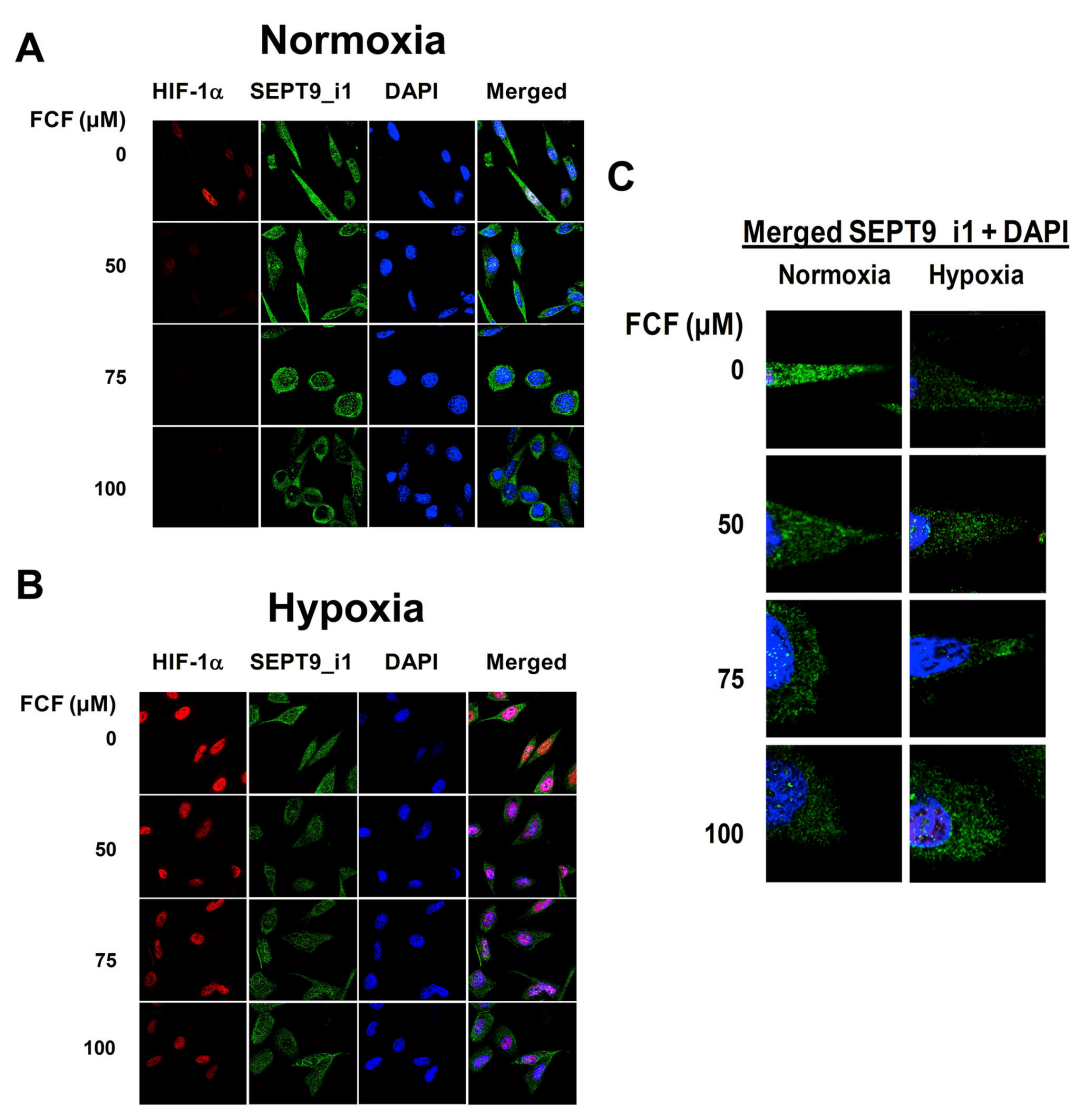
FCF alters SEPT9_i1 filamentous organization and localization. PC-3 cells were treated with FCF for 2 h and grown in normoxia (**A**) or hypoxia (**B**) for 4 additional h. They were fixed and processed for immunofluorescent labeling with anti-HIF-1α (red), anti-SEPT9_i1 (green) and DAPI (blue). Staining was analyzed by confocal laser-scanning microscope (magnification x63). (**C**) Representative cells from each treatment condition presented in (**A**) were further enlarged (zoom x2) for better visualization of SEPT9_i1 filaments (green).

We performed immunofluorescent staining in order to examine the effect of FCF on SEPT9_i1 filamentous organization and on HIF-1α localization (Figure 4). PC-3 cells were pre-incubated with increasing concentrations of FCF and then subjected to normoxia or hypoxia. Cells were fixed and immunostained for SEPT9_i1 and HIF-1α. As expected, HIF-1α expression was accumulated mainly in the nucleus following exposure to hypoxia (Figure 4A & B). Similar to Western blot analysis, FCF reduced HIF-1α protein levels under normoxia to a greater extent than under hypoxia (Figure 4A & B). In addition, FCF treatment changed cell morphology from a spindle-like shape to a round shape. It also changed SEPT9_i1 organization remarkably, from long structured filaments to shorter disrupted filaments (Figure 4C), and SEPT9_i1 protein localization was almost abolished from the nucleus and became more perinuclear or cytoplasmatic (Figure 4C). These findings indicate that FCF alters SEPT9_i1 filamentous organization and sub-cellular localization concomitant with HIF-1α inhibition.

### The effect of FCF on HIF-1α was general in cancer cells

**Figure 5:**
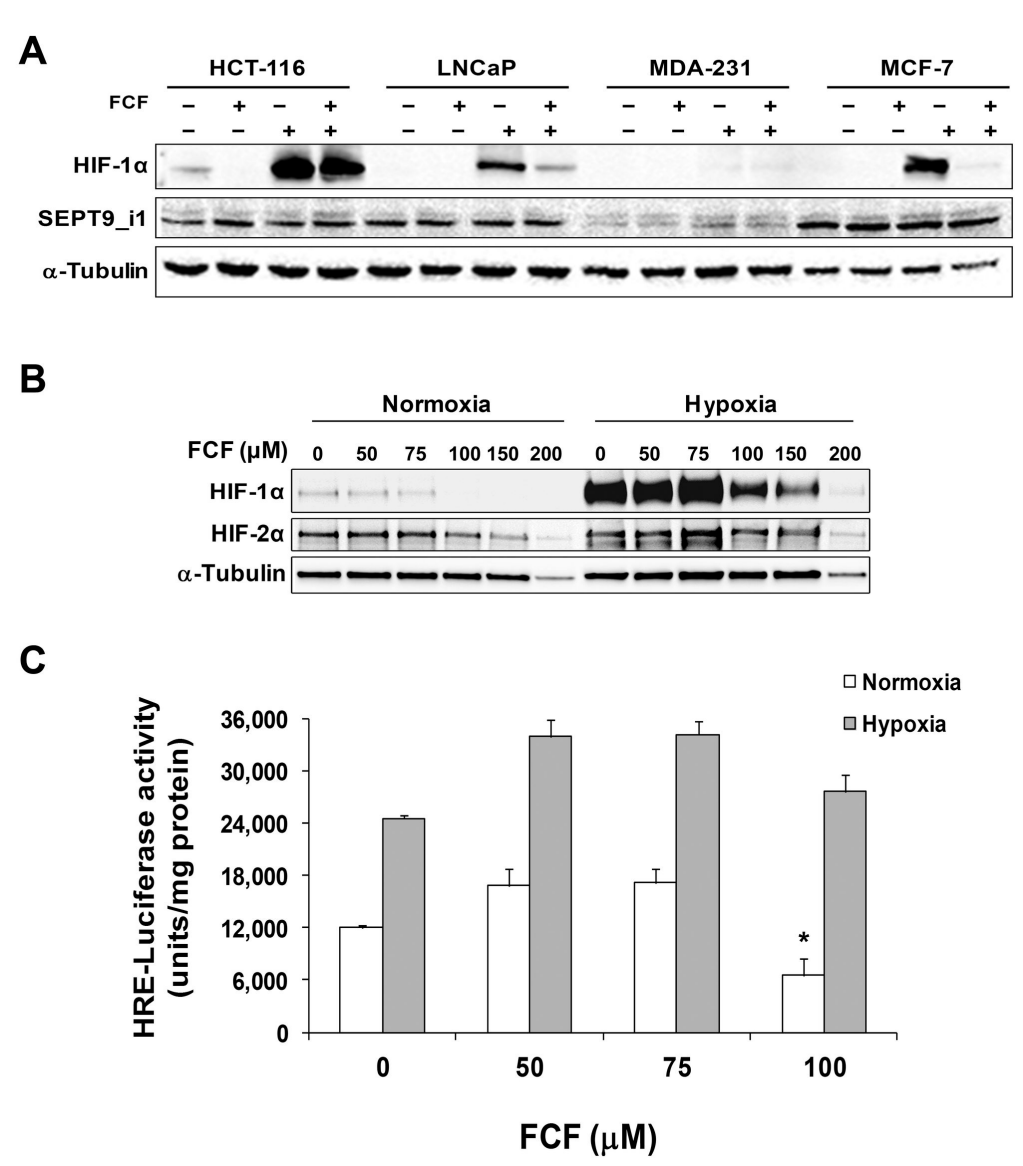
The effect of FCF on HIF-1α is general to cancer cells but specific only to HIF-1α. (**A**) The indicated cancer cells were treated with 100 μM FCF for 4 h under normoxia or hypoxia. Whole cellular extracts were subjected to Western blot analysis using anti-HIF-1α and anti-SEPT9_i1 antibodies. (**B**) PC-3 cells were treated with FCF as indicated and subjected to normoxia or hypoxia for 6 h. Whole cell extracts were analyzed by SDS-PAGE and immunoblotted with antibodies to HIF-1α, HIF-2α and tubulin. (**C**) PC-3 cells were transiently transfected with reporter plasmid expressing luciferase under the control of PTHrP P2 promoter (specific to HIF-2α). After 24 h of transfection, the cells were pretreated with FCF for 2 h and then subjected to normoxia or hypoxia for 48 h. Whole cell extracts were analyzed by luciferase luminescence assay. Arbitrary luciferase activity units were normalized to the amount of protein in each assay point. *Columns*, mean (n = 3); *bars*, SD. *P < 0.05.

Other cancer cells were tested to assess whether the inhibitory effects of FCF on HIF-1α are not restricted to PC-3 cells but are rather generalized. The effect of FCF on HIF-1α protein level was checked in prostate (LNCaP), colon (HCT-116) and breast (MCF-7, MDA-MB-231) cancer cells (Figure 5A). FCF reduced HIF-1α protein expression levels in most of the tested cancer cells. Of note, the most substantial reduction was obtained in cells that expressed the highest SEPT9_i1 protein levels (MCF-7), while no effect was observed in cells that barely expressed SEPT9_i1 (MDA-MB-231). These results revealed that the reduction of HIF-1α by FCF was a general occurrence in cancer cells and most likely dependent on SEPT9_i1 expression.

### The effects of FCF on HIF-2α expression and transcriptional activity

We characterized the specificity of FCF effect on HIF-α subunits by determining whether FCF would also affect HIF-2α. PC-3 cells were pre-incubated with FCF and cultured under normoxia or hypoxia (Figure 5B). HIF-2α protein expression levels were almost unaffected by FCF at 100 μM (IC_50_ >150 μM) under normoxic conditions, while the expression levels of HIF-1α were remarkably decreased (IC_50_ ~ 75 μM). We then examined HIF-2 transcriptional activity. HIF-1α protein is generally degraded over time under extended hypoxia, whereas HIF-2α is continuously accumulated [26]. We transiently transfected PC-3 cells with a reporter plasmid containing the luciferase gene under the control of HRE from the parathyroid hormone-related protein (*PTHrP*) P2 promoter. This promoter was discovered and characterized as a unique and direct target gene of HIF-2α in our previous work [21]. The transfected cells were treated with FCF for 48 h under normoxia or hypoxia (Figure 5C). There was no inhibitory effect of FCF on reporter activity under either normoxia or hypoxia, except for 100 μM FCF under normoxia (Figure 5C). It should be emphasized that FCF treatment was given for 48 h to test HIF-2 transcriptional activity compared to its having been given 16 h to measure HIF-1 transcriptional activity. Altogether, FCF was shown to have less significant inhibitory effects on HIF-2α protein expression levels and transcriptional activity compared to HIF-1α.

## Discussion

Homozygous deletion of *Sept9* in mice results in embryonic lethality, however, studies of *Sept9* embryonic fibroblasts confirmed the involvement of Sept9 in septin filament formation and overall cell stability [27]. The coexistence of different SEPT9 isoforms with various N-terminal extension affects septin heteromer polymerization composition and the ratio of hexamers to octamers for the higher-order arrangement of septin filaments [28]. We demonstrated that manipulating the dynamics of SEPT9_i1 filaments by FCF suppresses tumorigenic properties and downregulates both HIF-1α protein levels and HIF-1 transcriptional activity in cancer cells.

FCF influences not only SEPT9_i1 filaments but also disrupts other septin filaments [2,3,29]. Therefore, the effects of FCF on HIF-1α could be caused by general disruption of septin filamentous structures rather than disruption specific to SEPT9_i1-containing filaments. However, to the best of our knowledge, no interactions between HIF-1α and other septin family members have been described to date.

Septin filaments play a role in the stabilization of actin stress fibers [30,31]. It was also shown that SEPT9 filament formation in human mammary epithelial cells depends on interactions with microtubules (MTs) [32], and that other septins are implicated in the stability of MTs as well [4,33]. Moreover, Bowen et al. have recently found that septins provide a navigation mechanism for the growth and positioning of MTs by directing the longitudinal bundling of perinuclear MTs and the membrane targeting of peripheral MTs [34]. Interestingly, HIF-1α optimal function is tightly regulated by an intact MT-cytoskeleton structure [35–37]. Nevertheless, it is highly unlikely that the effects of FCF on HIF-1α are related to disruption of the MT/actin-cytoskeleton because FCF acts directly and specifically on the septin cytoskeleton and does not affect actin or MT assembly polymerization [3,29].

Our results showed that FCF did not alter HIF-1α mRNA levels but it did decrease HIF-1α protein stability and induce degradation rate (Figure 3), leading us to conclude that FCF downregulates HIF-1α protein at the posttranslational level. We had previously reported that the interaction between HIF-1α and SEPT9_i1 occurs mainly under normoxic conditions [12]. In agreement with those findings, the reduction of HIF-1α protein in the current study was considerably greater under normoxia compared to hypoxia, and the inhibition on HIF-1 transcriptional activity was also more substantial under normoxia (Figure 2). Furthermore, we previously reported that SEPT9_i1 interacts specifically with HIF-1α but not with HIF-2α [19]. We now showed that FCF has negligible effects on both HIF-2α protein levels as well as on HIF-2 transcriptional activity. We used the *PTHrP* P2 promoter that was discovered and characterized as a unique and direct target gene of HIF-2α in our previous work [21]. This promoter is being activated only after 48h of treatment and not after 16 h as in the HRE promoter (Figure 2C) or other downstream genes of HIF-1α. This is the reason why we could not directly compare between HIF-1 to HIF-2 activation in the same experiment. These results further confirm the hypothesis that FCF reduces HIF-1α expression and HIF-1 activation through disrupting SEPT9_i1 function. It is important to emphasize that these effects could be mediated by other indirect downstream yet unknown consequences of FCF.

It has been shown that FCF alters SEPT2 organization in HeLa cells [3]. Here, we show that FCF remarkably changed SEPT9_i1 organization and subcellular localization, i.e., from long structured to short filaments translocalized from the nucleus to the cell periphery (Figure 4). FCF disrupted HIF-1α/SEPT9_i1 interaction by increasing the interaction between the two proteins. Disturbance of the fine “thermodynamic” balance between free HIF-1α and HIF-1α complexed with SEPT9_i1 seems to be critical for optimal HIF-1α function. Septin family members are all assembled into stable homo- and heteromeric complexes, exhibiting a minimum of six subunits [28]. SEPT9 creates complexes with other human septin members, including SEPT2, 6, 7 and 11, and with the cytoskeletal proteins actin, tubulin and vimentin [38,39]. The role of these septin complexes and their cytoskeletal interactions in the activation of the HIF-1 pathway is yet unknown.

In summary, FCF inhibits the malignant potential of PC-3 cancer cells concomitantly with inhibition of the HIF-1 pathway and disruption of SEPT9_i1 filamentous structures. FCF or its modified derivatives may be potential new reagents for cancer therapeutics.
